# Effect of gamma radiation and accelerated aging on the mechanical and thermal behavior of HDPE/HA nano-composites for bone tissue regeneration

**DOI:** 10.1186/1475-925X-12-95

**Published:** 2013-09-24

**Authors:** Othman Y Alothman, Fahad N Almajhdi, H Fouad

**Affiliations:** 1 Department of Chemical Engineering, College of Engineering, King Saud University, Riyadh, Saudi Arabia; 2 Department of Botany and Microbiology, College of Science, King Saud University, Riyadh, Saudi Arabia; 3 Biomedical Engineering Department, Helwan University, Faculty of Engineering, Helwan, Egypt

**Keywords:** HDPE, HA, Nano-composite, DSC, DMA, Mechanical, Accelerated aging, Gamma radiation

## Abstract

**Background:**

The replacement of hard tissues demands biocompatible and sometimes bioactive materials with properties similar to those of bone. Nano-composites made of biocompatible polymers and bioactive inorganic nano particles such as HDPE/HA have attracted attention as permanent bone substitutes due to their excellent mechanical properties and biocompatibility.

**Method:**

The HDPE/HA nano-composite is prepared using melt blending at different HA loading ratios. For evaluation of the degradation by radiation, gamma rays of 35 kGy, and 70 kGy were used to irradiate the samples at room temperature in vacuum. The effects of accelerated ageing after gamma irradiation on morphological, mechanical and thermal properties of HDPE/HA nano-composites were measured.

**Results:**

In Vitro test results showed that the HDPE and all HDPE/HA nano-composites do not exhibit any cytotoxicity to WISH cell line. The results also indicated that the tensile properties of HDPE/HA nano-composite increased with increasing the HA content except fracture strain decreased. The dynamic mechanical analysis (DMA) results showed that the storage and loss moduli increased with increasing the HA ratio and the testing frequency. Finally, it is remarked that all properties of HDPE/HA is dependent on the irradiation dose and accelerated aging.

**Conclusion:**

Based on the experimental results, it is found that the addition of 10%, 20% and 30% HA increases the HDPE stiffness by 23%, 44 and 59% respectively. At the same time, the G’ increased from 2.25E11 MPa for neat HDPE to 4.7E11 MPa when 30% HA was added to the polymer matrix. Also, significant improvements in these properties have been observed due to irradiation. Finally, the overall properties of HDPE and its nano-composite properties significantly decreased due to aging and should be taken into consideration in the design of bone substitutes. It is attributed that the developed HDPE/HA nano-composites could be a good alternative material for bone tissue regeneration due to their acceptable properties.

## Introduction

Bone is one of the most commonly replaced tissues in the human body. Native bone tissues mainly consist of nonstoichiometric hydroxyapatite (HA; Ca_10_(PO_4_)6(OH)_2_) and collagen fiber matrix that provide physical and biological properties and mechanical support and protection from the vertebrate skeleton [1]. The growing demand for bone tissues replacement is due to the dramatic increase in patients suffering from bone cancer, traffic accidents and sport injuries. It is estimated that 4 millions operations carried out worldwide annually related to bone grafting or bone substitutes [2]. Currently, synthetic and natural origin materials such as metals, ceramics and polymers have been used for bone tissue engineering. Many studies have been carried out for developing different materials for bone tissue engineering such as metals, ceramics and polymers [1-5]. The metallic implants found some drawbacks such as high stress shielding and low corrosion resistance. The stress shielding is induced because the metallic bone substitute stiffness is far greater than the surrounding bone tissue. As a result, the surrounding bone tissues are insufficiently loaded compared to the metallic bone substitute leading to reduction in bone density [6,7]. The replacement of hard tissues demands biocompatible and sometimes bioactive materials with properties similar to those of bone. Nano-composites made of biocompatible polymers and bioactive inorganic nano particles have attracted attention as bone regeneration materials and even permanent bone substitutes due to their excellent mechanical properties and biocompatibility [8,9].

Composites that consisting of a polymeric matrix and bioactive micro/nano particles can provide new biomaterials for bone substitutes with acceptable mechanical and bioactive properties. The developed composite properties can be tailored for a given medical use according to the particles size and ratio. Several researchers reported work on developing HA/polymer based composites material for bone replacement [10-15]. Bonfield et al. introduced the bone-analogue concept by processing composite composed of a ductile polymer matrix (Polyethylene, PE) and stiff ceramic (HA) [10]. The mechanical coupling of the reinforcement and the matrix is resulting from the shrinkage of High density polyethylene (HDPE) matrix and HA particles during the composite processing [16]. The properties of HDPE/HA composite have been extensively studied by researchers [1,8,12,17,18]. The specific requirement for ideal materials for bone tissue regeneration is good mechanical properties, biocompatibility, and controlled resorbability [1]. There are two crucial factors play important role in producing HDPE/HA composites with bonelike properties. The first is good interfacial adhesion between organic polymers and inorganic HA. The second is the uniform dispersion of HA in the HDPE matrix. Kinga et al. [15] developed nano HA particles that have high surface area and fabricate HDPE/HA nano composite with superior mechanical properties similar to the mineral found in human hard tissues.

It is well known that HDPE/HA bone substitute has usually been irradiated for sterilization before implantation. The sterilization process promotes cross linking and changes the properties of the composite as a short term effect. On the other hand, the sterilization has negative effects on the enlargement density of long molecular chains as well as concentration of the tie molecules as a long term effect [19,20]. Also, it is well known that most of polymeric materials have a viscoelastic behavior even under normal loading conditions. Their overall mechanical properties change over time due to aging either on shelf or in vivo. Smolko and Romero [21] studied the effects of gamma irradiation dose on the properties of different types of polymeric matrix composites for application in hard-tissue replacement. The results indicated that the modulus of elasticity in tension and tensile strength increased with increasing the radiation dose while the fracture strain decreased. Albano et al. [22] studied the effect of gamma irradiation on properties of HDPE/HA composites prepared by solution. Results indicated strong increase in MFI with radiation dose while remarkable decrease in the crystallinity and melting temperature of HDPE/HA composites. Jin-Long et al. [23] studied the effects of HA percentage on the wear and mechanical behavior of ultra high molecular weight polyethylene (UHMWPE)/HA composites and results display enhancement in hardness, modulus and wear behavior of the composite with addition of HA. Fouad et al. [24] reported that viscoelastic, thermal, rheological, hardness, wear resistance properties, and fracture behavior of HDPE/HA composites changed due to accelerated thermal ageing.

It’s clear from literature review that little studies have investigated the effects of (accelerated or natural) aging after gamma irradiation on the behavior of HDPE/HA nano composites for biomedical applications. Therefore, the main objective of this part of study is to analyze the effects of different loading of nano HA particles, radiation and aging after radiation on the mechanical, thermal, visco-elastic and bioactivity behavior of the HDPE nano-composites and its application for bone tissue engineering.

## Materials and methods

### Materials

HDPE (melt index of 30 g/10 min; density of 0.954 g/cm^3^; and average molecular weight of 800000 g/mol) that procured by local Saudi manufacturer is used in the present study. It is an injection molding grade of HDPE copolymer with a narrow molecular weight distribution and high flow-ability. Spray dried synthetic HA nano particles with high purity and high surface area supplied by Fluidinova, Engenharia de Fluidos, SA, Portugal were used as reinforcement for HDPE matrix. The typical size of HA nano particle is less than 100 nm and the average aggregate size is 2.5 μm.

### Compounding

HDPE was premixed with different ratios of HA nano particles master-batch. The HA nano particles master-batch ratio was varied from 0 to 30%. Subsequently, the pre-mix was pelletized using an intermeshing and co-rotating twin screw extruder (Farrell FTX20) [24]. The granules were further dried and conditioned in the lab environment for 40 hours. The granules were then injection molded to get a set of standard ASTM D638 type-I specimens [25]. An injection molding machine (Asian Plastic Machinery, Double Toggle IM Machine, Super Master Series SM 120) was used to prepare ASTM standard samples. Although the standard recommends testing of at least five specimens, the ASTM standard allows for testing fewer specimens if so mentioned in the report. In the present study all the experimental tests were performed using three specimens. All the results presented here are the median of the three measurements.

### Gamma irradiation and aging procedures

The nano-composite specimens were exposed to gamma irradiation at doses of 0, 35 and 70 kGy at rate of 5 kGy/hr at room temperature in vacuum. The thermally accelerated aging of irradiated and non-irradiated HDPE/HA nano-composites were performed in saline solution at temperature of 80°C for 4 weeks that equals to 5 years of natural aging [24,26]. The aging procedure used in the present study is assumed to be resulted in an aging mechanism comparable to the real in vivo aging mechanism.

### Scanning electron microscopy (SEM)

The cryo-fracture surface of HDPE/HA nano-composites were examined using scanning electron microscope (SEM Model ISM 6360A, Jeol Company, Japan). The composite specimens are fixed with double coated carbon tape and surfaces of the samples were coated with a thin layer of gold under vacuum prior to the SEM observation to avoid electrostatic charging and heat build-up during observation.

### Cytotoxicity test

In vitro cytotoxicity tests were performed for HDPE and HDPE/HA composite specimens using the human amnion epithelium cell line known as Wistar Institute Susan Hayflick (WISH) cells monolayer [27]. The specimens were first sterilized in order to remove other microorganisms. Sterilization was carried out using gamma irradiation at doses of 70 kGy at rate of 5 kGy/hr at room temperature in vacuum. Each material was mixed with 10 ml RPMI medium supplemented with 10% fetal bovine serum, 1% MEM vitamins, 2 mM L-glutamine, 1000U penicillin, 1 mg streptomycin and 2.5 μg amphotericin-B was added to WISH cells monolayer for cytotoxicity evaluation. A separate flask of no HDPE specimen was used as control. All cultures were incubated at 37°C ± 1°C for 5 days and examined daily for any signs of cytotoxicity (cell degeneration, lysis and detachment) using an optical microscope.

### Tensile properties

The tensile properties of HDPE/HA composites were evaluated by using universal testing machine. The tensile tests up to fracture were performed at room temperature at speed of 5 mm/min. The output results were recorded as the applied load against elongation. At least three specimens were tested at each condition and the standard deviations are determined for all measured properties. The engineering yield strength was determined from the upper stress point, and Young’s modulus was calculated from the initial linear region of the stress–strain data.

### Differential scanning calorimetry (DSC)

Differential Scanning Calorimetry (DSC) tests of irradiated and non-irradiated HDPE and HDPE/HA nano-composites were performed on DSC-6 series (Schimadzu, Japan) with samples of ≈ 5 mg sealed in an aluminum pan. Each specimen was placed in the DSC oven and heated from 30°C /min to 200°C at rate of 5°C /min, then cooled down to 30°C at a cooling rate of 5°C /min. The heat of melting in each step was calculated by integrating the area under the DSC endothermic peak of the DSC thermogram. The melting temperatures of the samples were taken at the peaks of the melting processes. The percentage crystallinity was calculated by normalizing the heat of melting to that of 100% crystalline PE (290 J/g) [28-31].

### Dynamic mechanical analysis (DMA)

The visco-elastic properties of irradiated and non-irradiated HDPE and HDPE/HA nano-composite were characterized using a dynamic mechanical Analyzer (AR-G2 from TA, USA). The visco-elastic properties were measured under torsional arrangement. The samples were heated up to 80°C, stabilized at this temperature for 5 minutes, and then a frequency sweep test was performed. The frequency sweep was performed from 0.1 to 500 rad/sec.

## Results and discussions

### Morphological study

**Figure 1:**
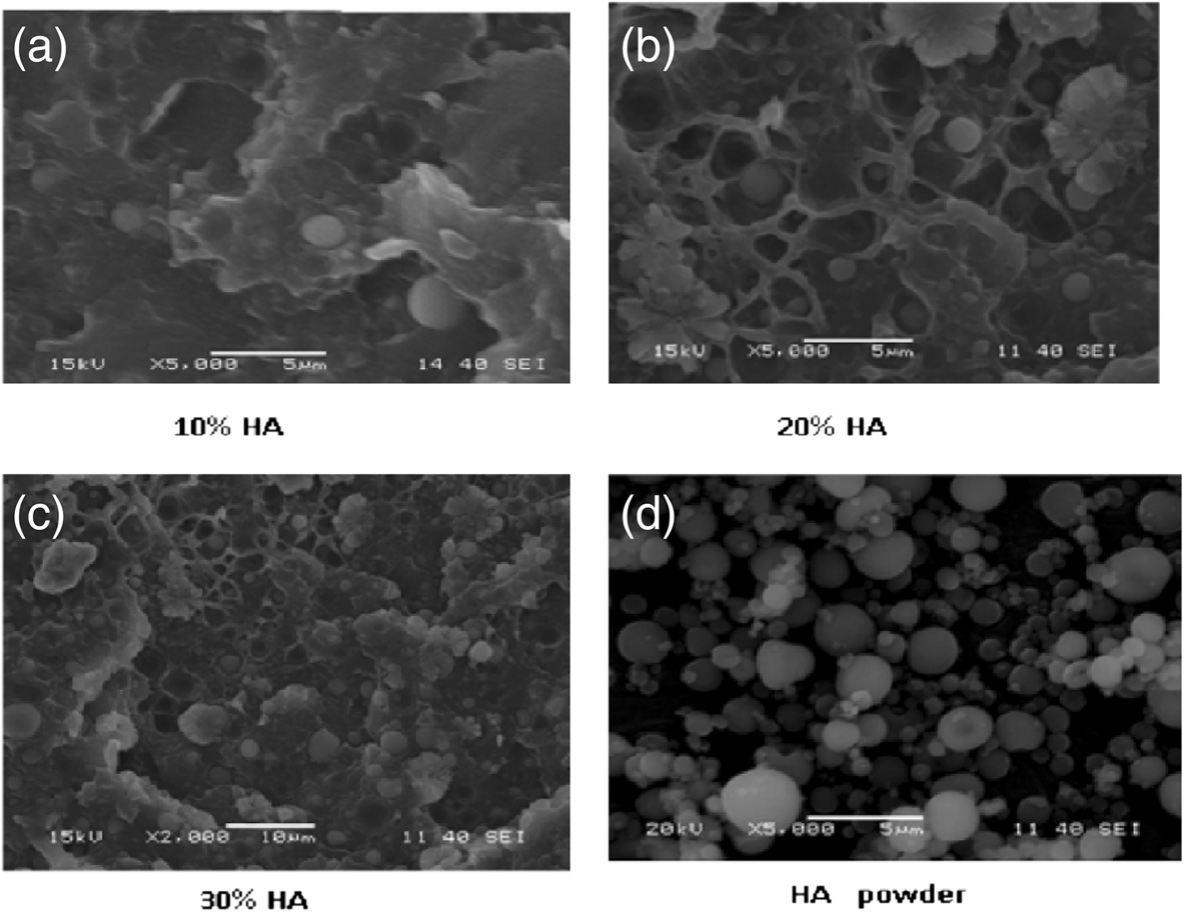
SEM micrographs of HDPE/HA nano-composites and neat HA powder: (a) 10 wt% HA, (b) 20 wt% HA, (c) 30 wt% HA and (d) HA powder.

The surface morphologies of HA powder and HDPE/HA composite specimens containing 10, 20 and 30 wt% HA are shown in Figure 1. It’s clearly indicated from SEM micrographs (Figure 1a-c) that the HA nanoparticles appear as bright spots distributed in the dark HDPE matrix. A reasonable distribution of HA particles was achieved in the HDPE/HA composites. Although, the HA particles were in the nano scale-range according to the manufacturer data but SEM micrographs display agglomeration of some nano particles to be in the micro scale range (100 nm). It is attributed that agglomeration of HA nano particles occurs due to the tendency of HA nano particles to decrease their contact surface with HDPE matrix. Therefore, bigger particle size of HA is clearly visible in the micrograph of HDPE/HA composite specimens.

### Cytotoxicity tests

**Figure 2:**
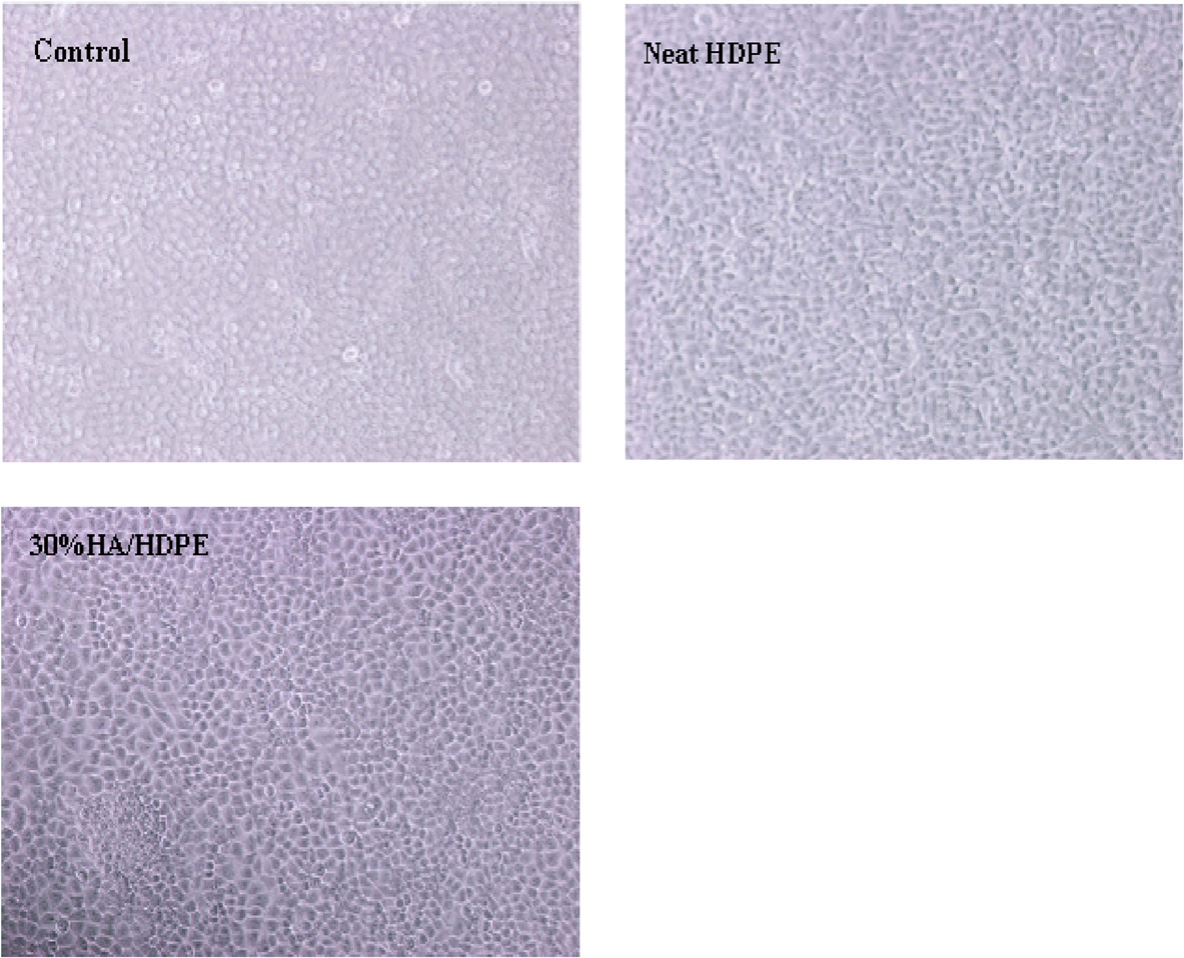
In vitro effect of HDPE and 30%HA/HDPE nano-composite specimens on WISH cells after 5 days.

The in vitro cytotoxicity tests of HDPE and HDPE/HA nano-composites showed that all the cells under experimental examination remained viable and retained the features of the normal cells until the end of the experiment duration. No distinct difference was noticed between the cultures containing neat HDPE, HDPE/HA nano-composites and the control test. Signs of cell lysis and/or degeneration were completely absent as shown in Figure 2. Thus, it can be concluded that the HDPE and HDPE/HA nano-composites do not exhibit any cytotoxicity to the WISH cell line. However, literature review reveals that experimental, histologic and clinical studies confirms the safety and efficiency of HDPE implants in aesthetic and reconstructive craniofacial surgery [30,32].

### Tensile properties

**Figure 3:**
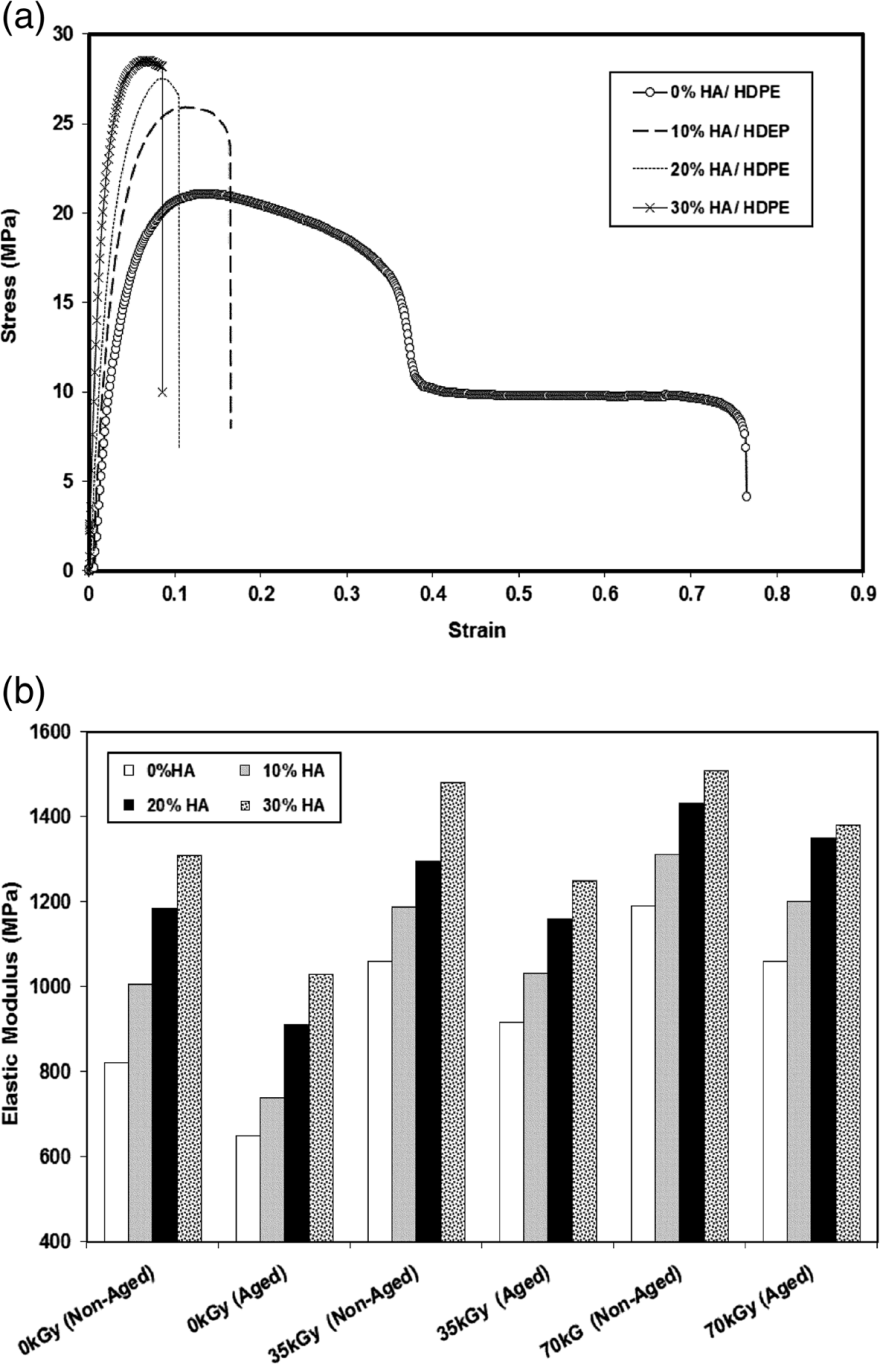
Effects of HA ratio on (a) Stress–strain behavior (b) Elastic Modulus of HDPE/HA nano-composites.

The strengthening effects of HA reinforcement rely on the homogeneity of HA nano particles dispersion in the HDPE matrix. HA nano particles dispersion in the HDPE polymer matrix resulted in enhancement of the mechanical properties of HDPE/HA nano composites. The results of tensile properties showed that the Young’s modulus of the neat HDPE and its nano composites are proportional to the HA content as indicated in Figures 3(a-b) and Table 1. The obtained results confirmed that the HA nano particles acted as reinforcement filler by transferring the sustaining load from the matrix to the rigid particles. From Table 1, it is found that the addition of 10%, 20% and 30% HA increases the HDPE stiffness by 23%, 44 and 59% respectively. It is believed that stiffness of HDPE/HA nano-composite increased due to the improvement of matrix stiffness and mobility restriction of polymer chains by incorporation of the HA nano-particles. Similar trends for the elastic modulus of HDPE/HA composite have been reported [16,18], where the modulus increased due to the addition of HA nano- particles to the HDPE matrix. Figure 3(b) illustrates the Young’s modulus as a function of irradiation dose and aging after irradiation of neat HA and HDPE/HA nano-composites. Young’s modulus of irradiated specimens showed remarkable increase of its value compared to non-irradiated ones due to the expected cross linking of HDPE polymer matrix that creates rigid regions that absorbed most of the applied load.

**Table 1:** Tensile properties of neat HDPE and HA/HDPE nano-composites

**Material**	**Non aged specimens**				**Aged specimens**		
	**Dose (kGy)**	**Modulus (MPa)**	**Ultimate stress (MPa)**	**Fracture strain**	**Modulus (MPa)**	**Ultimate stress (MPa)**	**Fracture strain**
**HDPE +0% HA**	0	820 ± 36	20.5 ± 0.8	76 ± 5.8	650 ± 38	18.3 ± 0.8	60 ± 4.9
	35	1060 ± 59	22 ± 1	48 ± 3.8	915 ± 55	22 ± 0.9	35 ± 3.1
	70	1190 ± 88	22.6 ± 0.9	26 ± 2.1	1059 ± 52	21.5 ± 1	22 ± 1.8
**HDPE + 10% HA**	0	1006 ± 58	25 ± 1.4	16.4 ± 1.1	740 ± 54	21 ± 1.1	13.3 ± 1.01
	35	1188 ± 79	24.6 ± 1.1	13 ± 0.92	1030 ± 60	23.9 ± 0.98	11 ± 0.87
	70	1310 ± 90	25.4 ± 0.95	10 ± 0.53	1200 ± 57	23.9 ± 1.1	9 ± 0.69
**HDPE + 20% HA**	0	1185 ± 67	27.5 ± 1.3	10.5 ± 0.58	910 ± 60	25.4 ± 1.6	9.3 ± 0.64
	35	1295 ± 70	28.1 ± 1.2	9 ± 0.72	1160 ± 63	26.9 ± 1.5	7 ± 0.48
	70	1430 ± 88	29 ± 1.7	8 ± 0.53	1350 ± 80	27 ± 1.2	7 ± 0.41
**HDPE + 30% HA**	0	1310 ± 81	28.8 ± 1.9	8.5 ± 0.41	1030 ± 69	26.3 ± 1.5	8.1 ± 0.58
	35	1480 ± 87	28.9 ± 1.5	7 ± 0.45	1250 ± 78	28 ± 1.6	6 ± 0.42
	70	1496 ± 89	29.1 ± 1.5	6 ± 0.39	1380 ± 81	27.5 ± 1.3	5 ± 0.33

**Figure 4:**
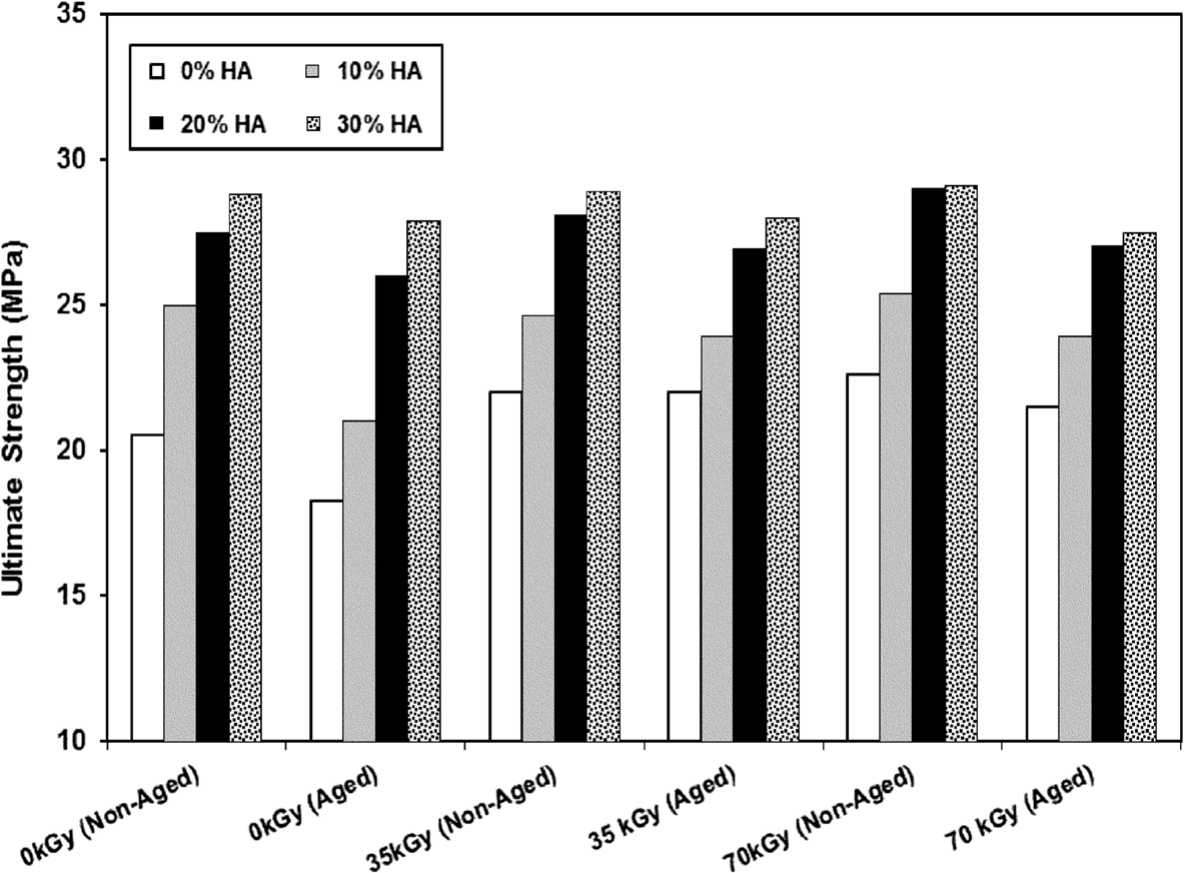
Variation of the ultimate strength for HDPE and HDPE/HA due to gamma irradiation and aging.

**Figure 5:**
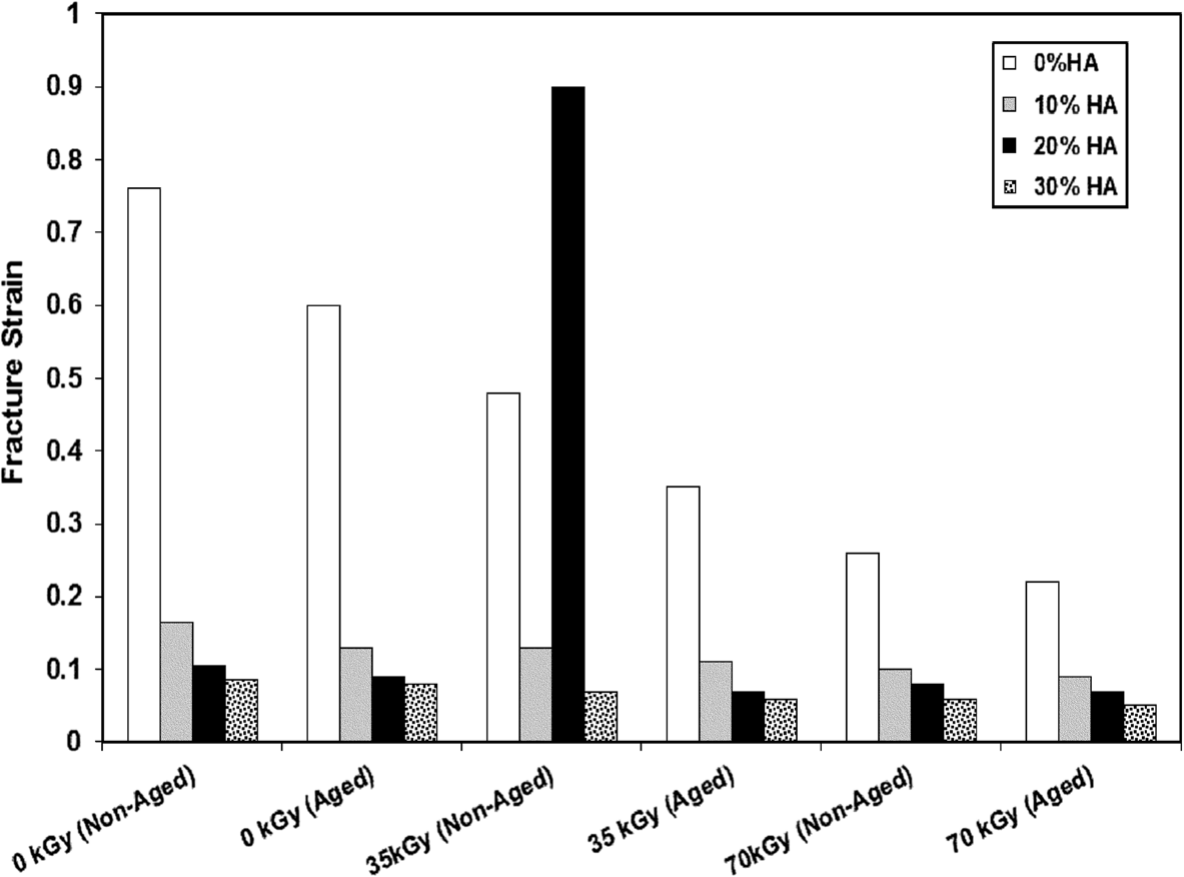
Variation of the fracture strain for HDPE and HDPE/HA due to gamma irradiation and aging.

Remarkable improvement in the tensile strength is observed for HDPE/HA nano composites compared to neat HDPE (Figure 4). Ultimate strength of HDPE/HA nano composites and neat HDPE shows an improvement with irradiation dose. Previous research reported that cavitations of the polymer matrix surrounding the rigid inorganic HA nano particles can promote extensive shear yielding which resulted in improvement of mechanical properties of polymers [30,31]. In an interested work, researchers reported that the tensile strength of HDPE/HA composite initially decreased with the addition of HA up to 20%, but increased for more than 30% HA [19]. Figure 5 shows the strain at fracture of the nano composites as a function of filler contents and irradiation doses. The fracture strain showed a strong decrease due to the addition of HA nano particles and gamma irradiation. These reduction in the fracture strain can be attributed to the cross linking of polymer amorphous region. The reduction of the fracture strain due to the addition of HA nano particles could be explained by the fact that the HA leads to a restriction of molecular mobility beside acting as a crack initiator or a stress riser in the nano composite leading to composite failure at low strains [19,30-32].

The accelerated aging of neat HDPE and HDPE/HA irradiated and non-irradiated nano composites resulted in remarkable changes in the behavior of the tested material. These changes are characterized by a decrease in the overall properties of the tested specimens (elastic modulus, material ductility and strength), compared with non-aged ones. The reduction in the tested materials properties due to aging can be attributed to the reduction in polymer chain segment by molecular chain session and the corresponding reduction of the tie molecules that would take place during accelerated aging [28-30]. It is important to mention that the reduction percentage in the overall mechanical properties of irradiated composite due to aging is lower than their values for non-irradiated ones. Obtained results display similar trends and agreement was observed between the present results and literature results [16-19,24,30,32].

### Differential scanning calorimetry (DSC)

The thermal behavior for the first heat (melting) of HDPE and HDPE/HA nano-composites with different HA percentage (wt%) are shown in Table 2. The crystallization of HDPE/HA nano composites was judged via the heat of fusion (ΔH), where the drop in heat of fusion indicates a decrease in the crystallization. The DSC results indicated that the crystallization decreased due to the presence of HA nano particles. From the results, it can be observed that the crystallinity of HDPE is 8% higher than that for HDPE/HA nano-composite (30%). The main reason of such drop is the formation of conglomerates of the filler. If these conglomerates exceed the critical size, they lose their nucleating capacity. It can be also attributed to the presence of HA particles that restrict the mobility of the molecules, hence the crystallinity decreases [30,31]. At such high HA contents it is obvious that degree of crystallinity decreases not only due to conglomeration of fillers but also due to e.g. confinement and entanglement effects. Moreover, the effect of nanofiller on polymer crystallization is strongly affected by the shape and concentration of nanoparticles [33]. On the other hand, it is known that the HA filler has higher specific heat capacity that make it better heat conductor resulting in faster cooling of HDPE/HA nano-composite. This high cooling rate resulted in thin lamellar formation rather than thick crystal growth leading to lower degree of crystallinity [30,31]. The DSC results also indicated that the melting temperature is slightly changed due to aging and gamma radiation.

**Table 2:** DSC results for aged and non aged HDPE and HA/HDPE composites

**Non aged specimens**							**Aged specimens**					
	**Xc% 0 kGy**	**Tm (°C)**	**Xc% 35 kGy**	**Tm (°C)**	**Xc% 70 kGy**	**Tm (°C)**	**Xc% 0 kGy**	**Tm (°C)**	**Xc% 35 kGy**	**Tm (°C)**	**Xc% 70 kGy**	**Tm (°C)**
HDPE + 0% HA	54 ± 1.8	134	60 ± 2.1	134.8	63 ± 1.8	135	59 ± 2.2	131.9	62 ± 2.2	132.1	63 ± 2.3	132.6
HDPE + 10% HA	51 ± 1.3	133	58 ± 2.1	133.2	59 ± 2	133.9	57 ± 1.8	131.6	59 ± 2.25	132.4	61 ± 2.25	132.8
HDPE + 20% HA	50 ± 1.31	132.3	54 ± 1.66	132.7	56 ± 1.6	133.2	52 ± 1.65	130.5	54 ± 1.58	131	58 ± 1.95	132
HDPE + 30% HA	44 ± 1	132.1	51 ± 1.2	132.5	53 ± 1.5	132.7	50 ± 1.6	130.4	52 ± 1.3	131.2	55 ± 1.9	132

**Figure 6:**
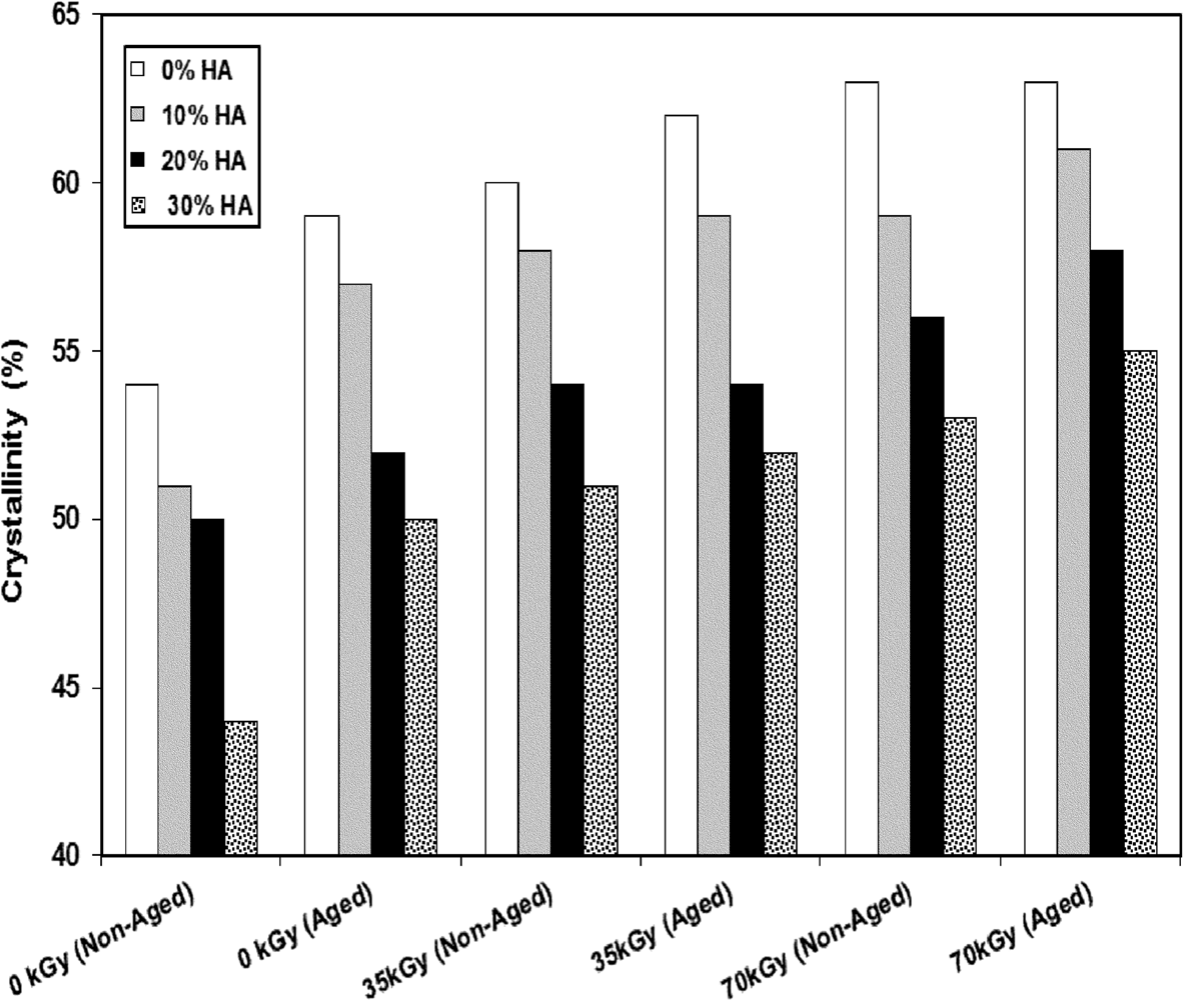
Variation in the crystallinity of HDPE and its composites due to irradiation and aging.

The results also, show that the crystallinity of HDPE and its nano-composite increases with increasing the irradiation dose as indicated in Figure 6. The crystallinity of neat HDPE and 30% wt HDPE/HA nano-composites increases by 9% due to irradiation with 70 kGy. Such increase of crystallinity can be attributed to the cross linking of polymer chains due to gamma irradiation. The DSC results of the first heat for accelerated aged HDPE and HDPE/HA nano-composites indicated that the crystallinity of accelerated aged specimens are about 6% higher than that of non-aged specimens for HDPE and its nano composites. These changes in the DSC results of aged HDPE and its nano composites can be attributed to the oxidation and chain scission of the tested material due to accelerated aging. The tested polymer chain scission process allows further crystal perfection and growth to occur. Therefore, the aged HDPE has higher degree of crystallinity compared to non-aged material [24,28,29].

### Dynamic mechanical analysis

**Figure 7:**
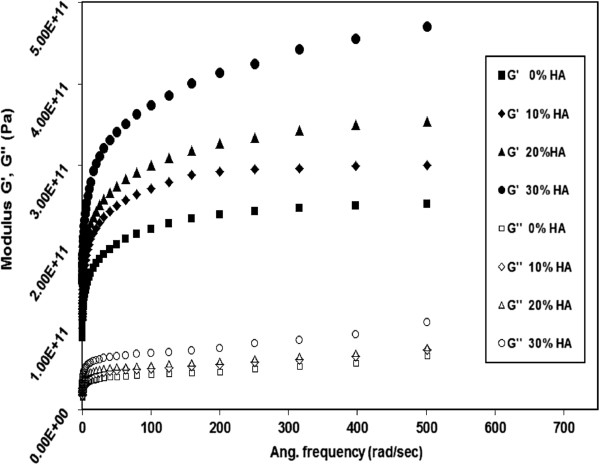
**Variation of G’ and G" with frequency for HDPE and HDPE nano composites at 80°C.**

The response of storage modulus (G’) and Loss modulus (G") to the testing frequency for neat HDPE and HDPE/HA nano-composites at 80°C are illustrated in Figure 7. The inset of Figure 7 shows G’ and G" for a wider range of frequency. For all tested materials, G’ and G" increased with increasing the testing frequency which is a manifestation of the viscoelastic behavior of HDPE and its nano-composites. It is noticed that G’ is more than G" for all materials which indicates that the elastic behavior of the material is dominant over the viscous one. It also noticed that G’ and G" of all nanocomposites increased with increasing the content of HA nano particles especially at high loading frequency. For example, at 500 rad/sec, the G’ increased from 2.25E11 MPa for neat HDPE to 4.7E11MPa when 30% HA was added to the polymer matrix. At the same testing conditions, G" for 30% HDPE/HA nano-composite was 60% higher than that of neat HDPE. The G’ and G" increase with HA content was also observed for the tensile results. This increase in the modulus of nano-composites can be attributed to the increase in the stiffness of polymeric matrix as a result of the decrease of free volume and the rise of mobility restriction due to the presence of HA nano-particles [30,31].

**Figure 8:**
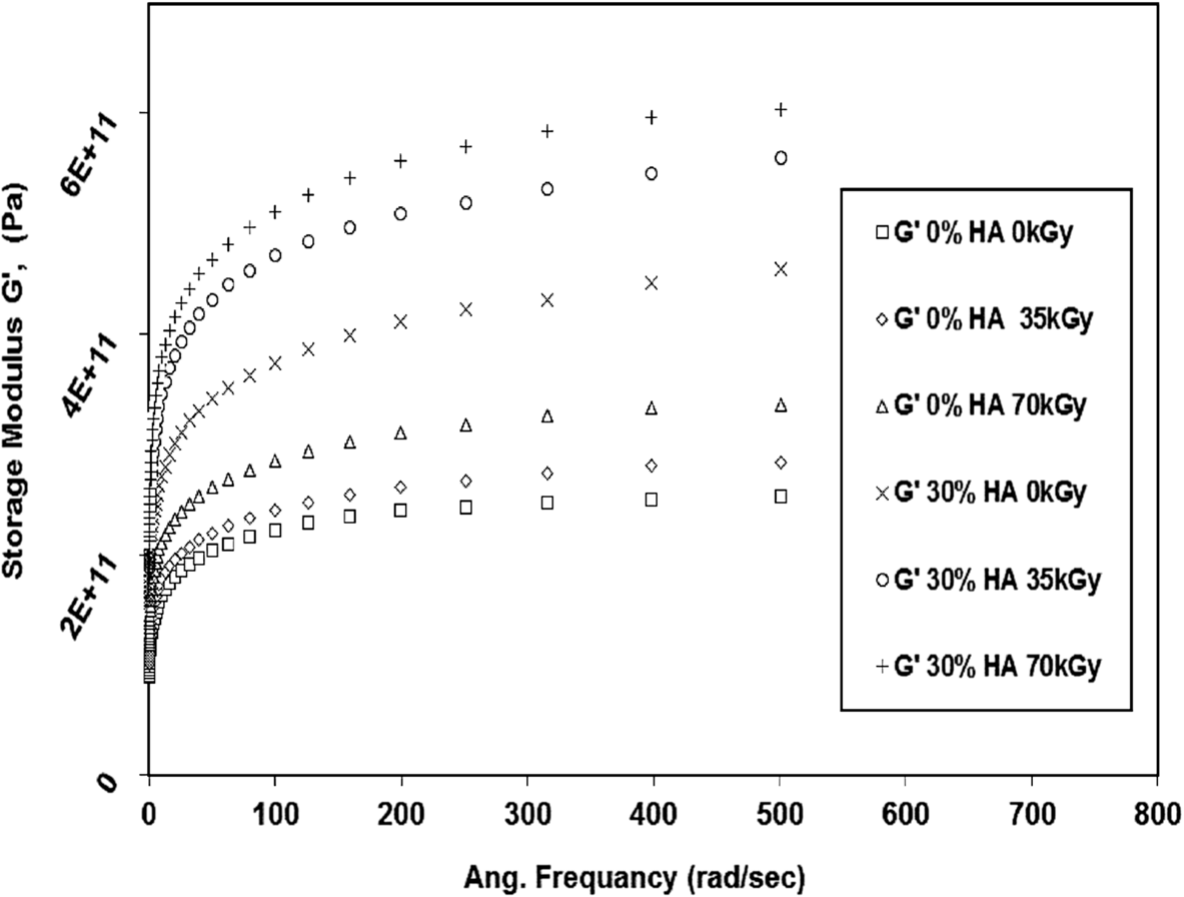
**Variation of G’ with frequency for irradiated HDPE and non-irradiated HDPE nano composites at 80°C.**

**Figure 9:**
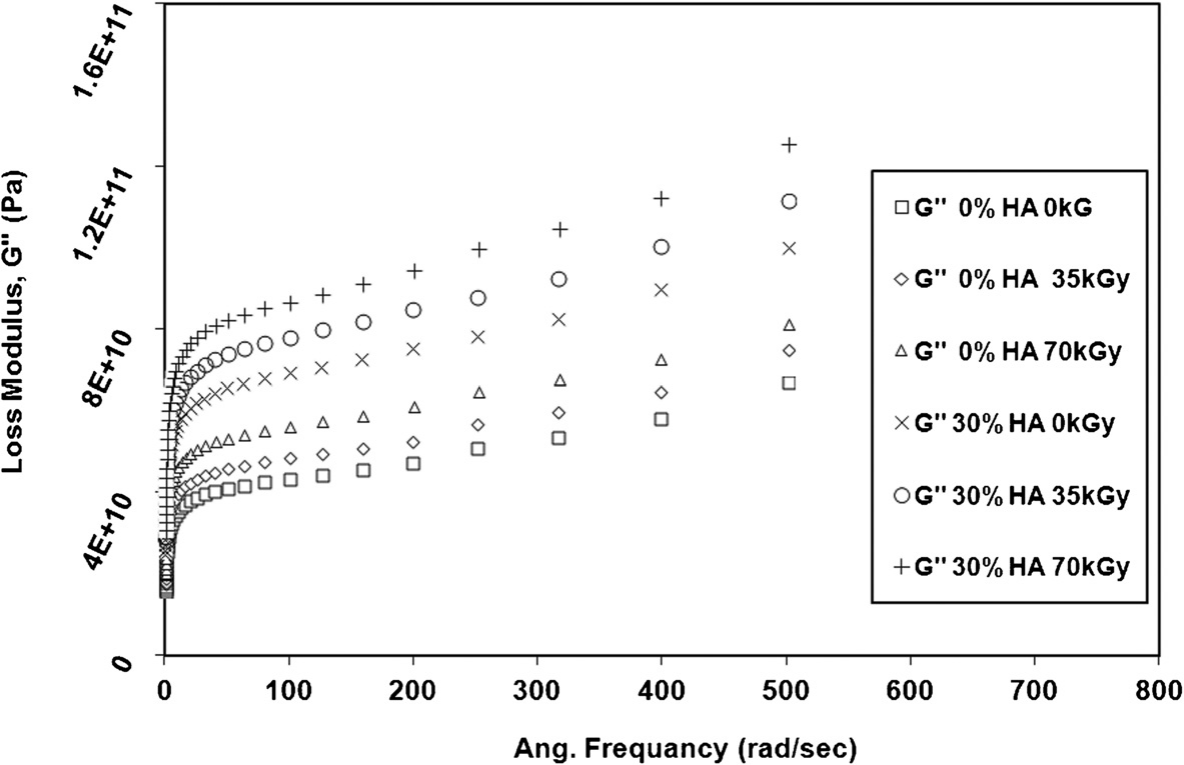
**Variation of G" with frequency for irradiated HDPE and HDPE nano composites at 80°C.**

The effects of gamma irradiation dose on the viscoelastic properties (G’ and G") of neat HDPE and 30% HDPE/HA nano-composites as a function of testing frequency are shown in Figures 8 and 9. The results showed that the G’ and G" significantly increased with increasing the irradiation dose. For example, at 500 rad/sec, the G’ for 30% HDPE/HA nano-composite increased by 22% and 32% due to irradiation with 35 kGy and 70 kGy, respectively. For the G’, at the same testing conditions, its value increased by 11% and 25% due to irradiation of 30% HDPE/HA nano-composite with 35 kGy and 70 kGy, respectively. Similar trends for G’ and G" have been obtained for the 10% and 20% HDPE/HA nano-composites. The improvement in modulus values due to irradiation can be attributed to the cross linking of polymer chains that is occur in the amorphous region and the creation of rigid regions in the polymer matrix.

**Figure 10:**
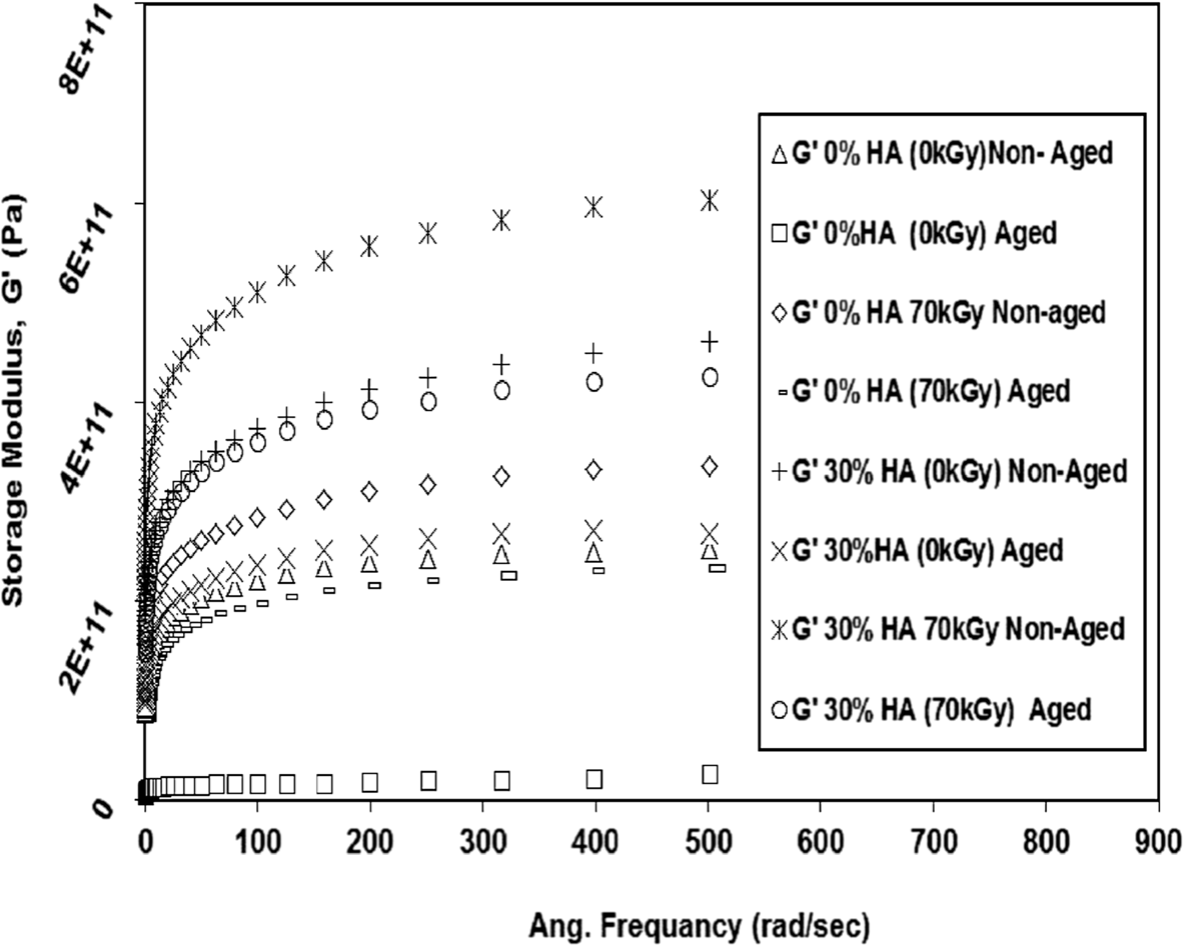
**Effect of aging on the G’ with frequency for irradiated and non-irradiated HDPE and HDPE nano composites at 80°C.**

**Figure 11:**
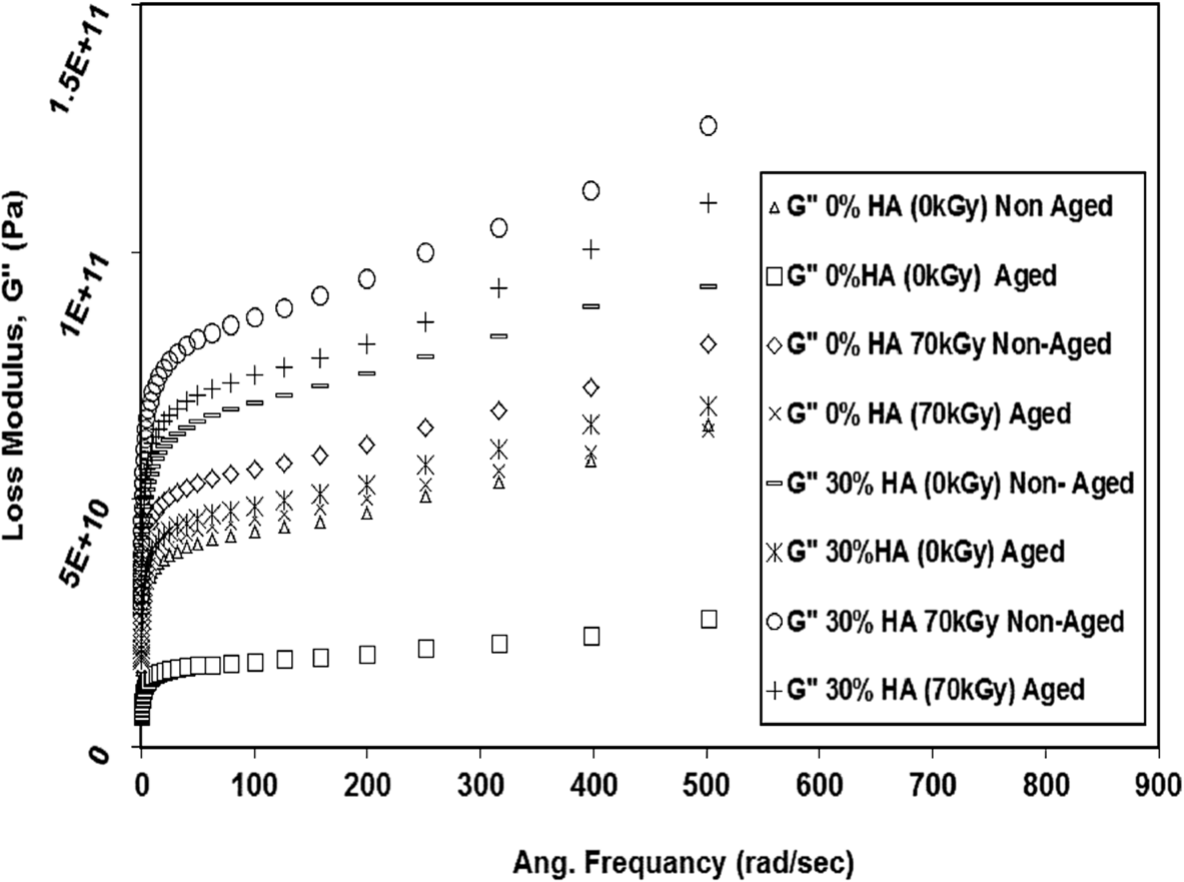
**Effect of aging on the G" with frequency for irradiated and non-irradiated HDPE and HDPE nano composites at 80°C.**

The accelerated aging of non irradiated and gamma irradiated specimens resulted in remarkable decrease in G’, and G" for all testing frequencies as indicated in Figures 10 and 11. The results showed that the G’ of non irradiated neat HDPE (0 kGy) decreased by 90% due to aging. This reduction in G’ due to aging becomes 30% for 70 kGy irradiated neat HDPE specimens. For 30% HDPE/HA (0 kGy), G’ decreased by 42% due to aging. For the same specimens (30% HDPE/HA) irradiated by 70 kGy, G’ dropped by 29%. Similar trends have been also observed for the G" where its values decreased due to aging for both irradiated and non irradiated neat HDPE and HDPE/HA nano-composites. The reduction in the properties of aged neat HDPE specimens and its nano-composites compared to non-aged ones are attributed to the material oxidation and its corresponding chain scission. The chain scission process results in an increase of polymer crystallinity (due to lamella thickening), breakage of the polymer long chains, reduction of tie molecules, and reduction in the tested material molecular weight, increasing material brittleness, and weakening their properties [20,24,28,29]. From the above results, it can be remarked that the reduction in the properties of non-irradiated HDPE and HDPE/HA nano-composites due to aging is higher than that for the irradiated ones. This may be attributed to that the cross linking due to irradiation is predominant than the chain scission process which occurred due to aging.

From the losses modulus (DMA) results, it can be noticed that the glass transition temperature (Tg) ranges from 31°C to 33°C for irradiated and non-irradiated neat HDPE and HDPE/HA nano composites. According to these results, it can be concluded that there is no significant change in Tg due to the presence of HA or due to gamma irradiations. Also, it is important to mention that the values of Tg weren’t clear in our DSC graphs.

## Conclusions

HDPE with good distribution of HA nano particles was successfully prepared using twin screw extrusion. Neat HDPE and HDPE/HA nano-composites do not exhibit any cytotoxicity to the WISH cell line. HA nano particles decreased the crystallinity of HDPE composites due to the restriction of mobility of the molecules. Aging of HDPE/HA nano composites resulted in increase of the crystallinity due to expected oxidation and chain scission. With increasing the HA nano particles contents, the storage, loss modulus and Young’s modulus of the nano-composite increased while the strain at fracture decreased proportional to the HA content. Irradiation of HDPE and HDPE/HA nano composites resulted in significant modification in their mechanical and visco-elastic behavior. Aging of HDPE/HA nano-composites resulted in decrease of the storage, loss modulus, Young’s modulus and strain at fracture. The reduction in the properties of non-irradiated HDPE and HDPE/HA nano-composites is due to aging is higher than that for the irradiated ones. HDPE/HA nano-composite could be a good alternative materials for bone tissue regeneration. Finally, it is attributed that the developed HDPE/HA nano-composites could be a good alternative advanced materials for bone tissue regeneration due to their acceptable architectures and properties.

## Competing interests

The authors certify that there is no actual or potential conflict of interest to declare in relation to this paper.

## Authors’ contributions

FNA was involved in the design of study from the biological point of view, save the biological materials as well as helped in the manuscript preparation. HF and OYA were involved in the design of study from the engineering point of view, fabrication and characterization of the composite under investigation as well as drafted the manuscript. All the authors have read and approved the final manuscript.
